# Prognosis and treatment effects of HIV-associated talaromycosis in a real-world patient cohort

**DOI:** 10.1093/mmy/myab005

**Published:** 2021-02-27

**Authors:** Jonathan Klus, Vo Trieu Ly, Cliburn Chan, Thuy Le

**Affiliations:** Department of Statistical Science, Duke University, Durham, NC 27708, USA; Department of Infectious Diseases, University of Medicine and Pharmacy at Ho Chi Minh City, Ho Chi Minh City, Vietnam; Hospital for Tropical Diseases, Ho Chi Minh City, Vietnam; Department of Biostatistics and Bioinformatics, Duke University School of Medicine, Durham, NC 27710, USA; Oxford University Clinical Research Unit, Ho Chi Minh City, Vietnam; Division of Infectious Diseases and International Health, Duke University School of Medicine, Durham, NC 27710, USA

**Keywords:** talaromycosis, penicilliosis, *Talaromyces marneffei*, HIV, prognosis

## Abstract

Talaromycosis is a leading cause of AIDS-associated opportunistic infections and death in Southeast Asia. We have recently shown in the Itraconazole versus Amphotericin for Talaromycosis (IVAP) trial that induction therapy with amphotericin B reduced mortality over 24 weeks, but not during the first 2 weeks. Antifungal treatment effects in real-world settings have not been rigorously evaluated. Using data obtained from patient records at the Hospital for Tropical Diseases, Ho Chi Minh City, Vietnam from 2004 to 2009, we first developed a prognostic model using Bayesian logistic regression to identify predictors of death. Second, we developed a causal model using propensity score matching to assess the treatment effects of amphotericin B and itraconazole.

Our prognostic model identified intravenous drug use (odds ratio [OR] = 2.01), higher respiratory rate (OR = 1.12), higher absolute lymphocyte count (OR = 1.62), a concurrent respiratory infection (OR = 1.67) or central nervous system infection (OR = 2.66) as independent predictors of death. Fever (OR = 0.56) was a protective factor. Our prognostic model exhibits good in-sample performance and out-of-sample validation, with a discrimination power of 0.85 and 0.91, respectively. Our causal model showed no significant difference in treatment outcomes between amphotericin B and itraconazole over the first 2 weeks (95% credible interval: 0.62, 2.50).

Our prognostic model provides a simple tool based on routinely collected clinical data to predict individual patient outcome. Our causal model shows similar results to the IVAP trial at 2 weeks, demonstrating an agreement between real-world data and clinical trial data.

## Introduction

*Talaromyces marneffei* causes an invasive fungal infection called talaromycosis (formerly penicilliosis) in immunocompromised residents and travelers to Southeast Asia, particularly in people with advanced HIV disease.^1^^,^^2^ In just over two decades, talaromycosis cases have surged from a rare infection to among the most common HIV-associated infections in highly endemic countries including Vietnam, Thailand and China.^3–5^ The prevalence among HIV hospital admissions ranges between 4 and 16%.^1^^,^^4^^,^^6^^,^^7^ Despite this high disease prevalence and a mortality of up to 33% on antifungal therapy,^1^^,^^4–7^ clinical factors driving disease severity and patient outcomes remain poorly understood.

Although international guidelines recommend induction therapy with amphotericin B, itraconazole is used more frequently due to better availability, tolerability and lower cost in lower-income countries in Asia.^1^^,^^4^^,^^5^^,^^6^^,^^8^^,^^9^ In a landmark multi-center randomized control trial (RCT) of Itraconazole versus Amphotericin B for Talaromycosis (formerly Penicilliosis) (IVAP, N = 440 patients recruited between 2012 and 2015), we have demonstrated that induction therapy with amphotericin B was superior to itraconazole in mortality over 24 weeks (11.3 versus 21.0%, absolute risk difference of 9.7 percentage point, 95% confidence interval: 2.8–16.6; *P *= 0.006). However, a mortality benefit was not observed in the first 2 weeks.^10^ While RCTs are the gold standard for outcomes research, they often exclude important subgroups of patients with comorbidities. The IVAP trial excluded patients who were deemed unsafe to receive amphotericin (e.g., creatine clearance <30 ml/min) or to itraconazole (e.g., AST or ALT > 400 units/l, on concurrent rifampicin, or pregnancy), limiting the generalizability of treatment effects in excluded patients. Antifungal treatment effects in real-world settings have not been rigorously evaluated.

We previously published a retrospective study of 513 patients with culture-confirmed talaromycosis at the Hospital for Tropical Diseases (HTD) in Ho Chi Minh City. In this real-world patient cohort, induction therapy with itraconazole (62%) or amphotericin B (36%) was driven by the patient's ability to pay under Vietnam's fee-for-service healthcare system. The clinical and laboratory characteristics on admission, antifungal treatments and patient outcomes at the time of hospital discharge were systematically analyzed.^1^ The goals of this study are to (i) develop and validate a prognostic model to assist clinicians in identifying patients at initial presentation who are at higher risk of a poor outcome, and thus may require a more aggressive management approach; (ii) use causal inference techniques to assess outcomes in patients treated with amphotericin B and itraconazole to evaluate the generalizability of the IVAP trial results.

## Methods

### Ethics statement

This study was approved by the ethics and scientific committee of the HTD in Ho Chi Minh City, Vietnam (Study number: CS/NĐ/09/19).

### Data source

Medical records were obtained from the HTD for 513 unique patients treated from 2004 to 2009.^1^ Inclusion criteria were: (i) age ≥18 years, (ii) culture-confirmed talaromycosis from blood, skin lesions or any sterile site, (iii) an assessable outcome at discharge. A total of 489 patients in the dataset met these criteria. We defined poor outcomes as patients who died in the hospital or were discharged home to die in deteriorating condition. Good outcomes were defined as patients who were discharged with resolution of fever, improving skin lesions and/or clearance of follow-up cultures. Patients for whom outcomes were classified as non-assessable were excluded from this analysis. After initial cleaning, the data were randomly split into a training set (75% of sample, n = 367) and a hold-out set (25% of sample, n = 122) to allow for out-of-sample validation at the conclusion of the model development. All data were analyzed using R version 3.5.1.^11^

### Prognostic model development

To assess candidate predictors that might be associated with patient outcomes, we consulted existing literature on prognostic models, including the Acute Physiology and Chronic Health

Evaluation (APACHE) IV score for ICU patients,^12^ as well as the existing talaromycosis literature.^1^^,^^4^^,^^6^^,^^13–16^ The following baseline characteristics were included in the model: sex, intravenous drug use (IVDU), fever ≥38°C, skin lesions characteristic of talaromycosis, pulse rate, respiratory rate, and certain laboratory results. Also included in the model were concurrent opportunistic infections that were clinically diagnosed at the time of patient evaluation. This included syndromic diagnoses of pneumonia (including *Pneumocystis jirovecii* pneumonia, pulmonary tuberculosis) or central nervous system (CNS) infections (including cryptococcal meningitis, cerebral toxoplasmosis). The prognostic model was developed blind to treatment assignment or duration. Prior to model estimation, each covariate was explored by independently assessing their relationship with patient outcomes in the training data. Predictors that contained missing values were imputed using the Multiple Imputation by Chained Equations (MICE) package.^17^ Fifty imputed datasets were generated using partial means matching for continuous predictors and logistic regression for binary predictors. Modeling was performed using a Bayesian multivariate logistic regression model. The model was fit with Bayesian Regression Models using Stan (BRMS) version 2.9.0,^18^ a front-end interface for the Stan probabilistic programming language.^19^ We ran four chains of 10 000 iterations each, with a warm-up of 1000 iterations. A normal prior with a mean of 0 and standard deviation of 0.5 was placed on the model coefficients.

### Prognostic model validation

As stressed by Royston^20^ and others in the statistics and medical literature, validation using data not included in the training set is necessary to ensure the robustness of the model. We performed both in-sample and out-of-sample validation in order to assess model performance. The methods employed included the area under the receiver operating characteristic (AUROC) curve that is typically used to assess classification performance,^21^ as well as the calibration curve suggested by Altman^22^ and the APACHE IV score authors.^12^

### Causal model development

Patients in this cohort were not randomly assigned to antifungal drugs. To control for potential bias in the sample due to non-random assignment, we used a causal modeling approach. The data used for the causal model was limited only to those who received at least two days of antifungal treatment, and received either amphotericin B followed by itraconazole, or received itraconazole therapy without switching to amphotericin mid-cycle. We used the propensity score matching technique initially developed by Rosenbaum and Rubin.^23^^,^^24^ In this analysis, bias reduction would be indicated by a decrease in the standardized mean difference (SMD) for covariates that were imbalanced in the original data. Generally speaking, an SMD of less than 0.1 is the heuristic used to indicate acceptable balance for a given covariate.^25^ Unlike the prognostic model, the goal was not to build a model with good out-of-sample predictive capability, but to assess the effect that antifungal treatment had on patient outcomes using observational data.

In order to engineer a cohort that would emulate the design of a clinical trial, the propensity for treatment assignment was modeled using logistic regression via the R package MatchIt.^26^ One-to-one matching was used to create a study population matched on the same covariates used for the prognostic model, with the addition of age and some additional baseline laboratory results. This matched sample was then modeled using a similar technique as described for the prognostic model, with the addition of the treatment variable.

## Results

### Prognostic model results

Table 1 shows the baseline characteristics of 367 patients included in the training dataset and adjusted odds ratios (OR) for poor outcomes using the Bayesian multivariate logistic regression model. The majority of patients were male, had a history of IVDU, had characteristic skin lesions and had fungemia. Patients with poor outcomes had a higher prevalence of IVDU (81 versus 64%), a higher respiratory rate (31.4 breaths/min versus 25.4 breaths/min), a higher prevalence of comorbid lower respiratory infection (43 versus 28%) or CNS infection (23 versus 2%), but a lower prevalence of skin lesions (64 versus 73%) and fever (27 versus 34%).

**Table 1.** Baseline characteristics and predictors of poor outcomes at day 30 for 367 patients with HIV-associated talaromycosis included in the training dataset.

	Good outcomes n = 261	Poor outcomes n = 106	Odds ratio	2.5% (lower)	97.5% (upper)
Sex (male)	0.81	0.88	1.12	0.59	2.17
**IVDU (yes)**	**0.64**	**0.81**	**2.01**	**1.14**	**3.56**
**Fever (>38°C)**	**0.34**	**0.27**	**0.56**	**0.32**	**0.98**
Pulse (beats/minute)	99.39	104.06	1.00	0.98	1.02
**Respiratory rate (breaths/minute)**	**25.41**	**31.44**	**1.12**	**1.07**	**1.17**
Skin lesions (yes)	0.73	0.64	0.78	0.46	1.32
**Absolute lymphocyte count (cells/μl)**	**0.49**	**1.02**	**1.62**	**1.11**	**2.44**
Hemoglobin (g/dl)	7.73	7.87	0.98	0.88	1.10
Platelets (10^3^/μl)	127.76	86.07	1.00	0.99	1.00
**Creatinine (10 μmol/l)**	**9.02**	**15.90**	**1.08**	**1.04**	**1.14**
**AST (10 units/l)**	**15.19**	**26.13**	**1.02**	**1.01**	**1.04**
**Lower respiratory infection (yes)**	**0.28**	**0.43**	**1.67**	**1.01**	**2.75**
**CNS infection (yes)**	**0.02**	**0.23**	**2.66**	**1.30**	**5.48**
Fungemia (yes)	0.93	0.96	1.23	0.55	2.80

*Note*: Summary statistics are presented as count (percentage) of positive value for categorical variables and mean for continuous variables. Odds ratios and upper and lower bounds for 95% credible intervals (CI) are estimated from the Bayesian multivariate logistic regression model, and were therefore adjusted by the model covariates. AST: aspartate aminotransferase, CNS: central nervous system, IVDU: intravenous drug use.

The predictors of poor outcomes in the multivariate model included IVDU (OR = 2.01, 95% CI: 1.14–3.56), higher respiratory rates (OR = 1.12, 95% CI: 1.07–1.17), higher absolute lymphocyte counts (OR = 1.62, 95% CI: 1.11–2.44), 10-unit increase in aspartate aminotransferase (AST) levels (OR = 1.02, 95% CI: 1.01–1.04), a 10-unit increase in creatinine levels (OR = 1.08, 95% CI: 1.04, 1.14), a concurrent lower respiratory infection (OR = 1.67, 95% CI: 1.01--2.75) or a CNS infection (OR = 2.66, 95% CI: 1.30–5.48). Fever was associated with decreased odds of poor outcomes (OR = 0.56, 95% CI = 0.32–0.98).

**Figure 1.**
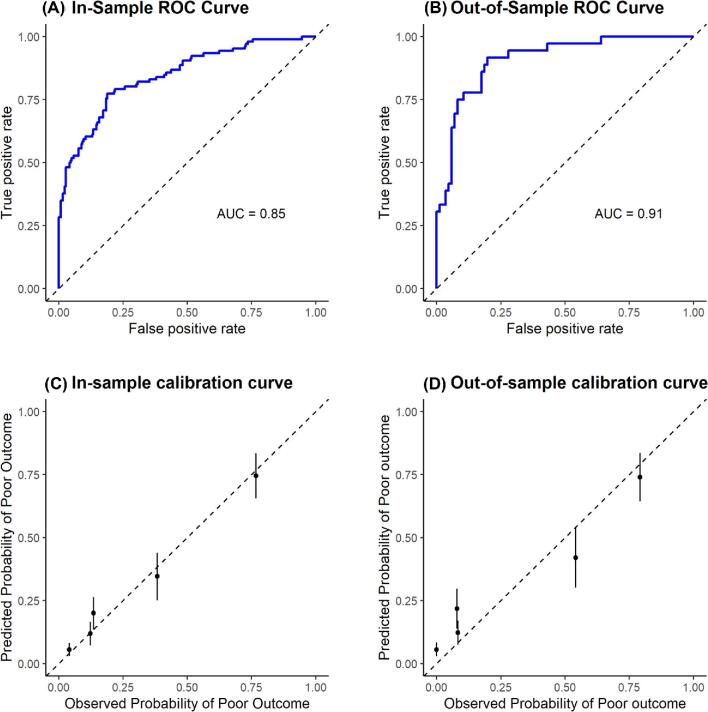
(A, B) legend: The receiver-operator characteristic (ROC) curves for the prognostic model. The ROC curves had an area under the curve of 0.85 in-sample and 0.91 out-of-sample, indicating good discrimination between good and poor outcomes. (C, D) legend: The calibration curves for the prognostic model. Predicted probabilities of poor outcomes were split into five risk quintiles and plotted against observed probability of poor outcomes in that quintile to determine model calibration to observed risks. The points that fall along the dashed 45-degree line indicate perfect agreement between the predicted probability and the observed probability of poor outcomes in the data. Error bars are ± 1 standard deviation of draws from the posterior distribution of the outcome. While agreement between the predicted and observed data appears strong for lower-risk quintiles, there were some deviations as the observed probability of poor outcomes increased.

The prognostic model had a discriminatory power (AUROC) of 0.85 in-sample, and 0.91 out-of-sample (Fig. 1A, B). The in-sample and out-of-sample calibration curves show good calibration between predicted and observed probabilities of poor outcomes (Fig. 1C, D). While agreement appears strong for lower-risk quintiles, there were some deviations as the observed probability of poor outcomes increased.

### Causal model results

The causal model employed more stringent inclusion criteria than the prognostic model in order to assess the treatment effect. From the original 513 patients considered for this study, we eliminated patients under age 18 (n = 6); patients who had died before cultures became positive, hence did not receive antifungal therapy (n = 69); and patients who had a non-assessable outcome (n = 18). To engineer a cohort more compatible with the IVAP trial, we further eliminated patients who were hospitalized for less than two days (i.e., too short to assess treatment effect, n = 45); those who received less than 7 days of amphotericin or itraconazole due to intolerance, death or deteriorating condition (n = 115); and those who required a switch from itraconazole to amphotericin due to treatment failure (n = 51). The covariate balance for 421 patients when the main exclusion criteria were applied and for the 242 patients when additional IVAP exclusion criteria were applied are reported in Tables S1 and S2, respectively. Note that these additional exclusion criteria were not applied to the prognostic model, which was developed blind to treatment assignment and duration.

Table 2 shows the effect of applying propensity score matching using logistic regression on the covariate balance for the 242 patients when additional IVAP exclusion criteria were applied. The SMD for most of the baseline variables was less than 0.1, except for IVDU, history of tuberculosis, weight, length of stay, aspartate aminotransferase (AST), systolic blood pressure, skin lesions and respiratory rate. A sensitivity analysis was conducted for the 421 patients when the main exclusion criteria were applied; the results are reported in Supplementary Table 3. Implementing a caliper of 0.2 standard deviations decreased the number of covariates for which the SMD exceeded 0.1 but at the expense of a reduced sample size (Supplementary Table 4). The distributions of matched and unmatched propensity scores following application of the matching procedure for the 242 patients when additional IVAP exclusion criteria were applied and for the 421 patients when main eligibility criteria were applied are shown in Supplementary Figure 1.

**Table 2.** Summary statistics for amphotericin B and itraconazole treatment groups following propensity score matching (n = 242).

	Amphotericin B	Itraconazole	SMD
N	70	70	–
Age (years)	28.79 (4.71)	28.66 (6.00)	0.02
Sex (male)	54 (77.1)	56 (80.0)	0.07
IVDU (yes)	40 (57.1)	46 (65.7)	0.18
History of TB (yes)	23 (32.9)	18 (25.7)	0.16
History of TM (yes)	5 (7.1)	5 (7.1)	<0.001
Fever (>38**°C**)	26 (37.1)	24 (34.3)	0.06
Pulse (beats/min)	97.63 (16.51)	97.29 (15.71)	0.02
SBP (mm/Hg)	100.14 (11.73)	98.79 (10.44)	0.12
Respiratory rate (breaths/minute)	25.74 (5.10)	24.74 (4.95)	0.20
Weight (kg)	44.61 (7.64)	42.80 (7.06)	0.25
Skin lesions (yes)	31 (44.3)	49 (70.0)	0.54
Absolute lymphocyte count (cells/μl)	0.59 (0.93)	0.68 (0.86)	0.10
Hemoglobin (g/dl)	8.14 (2.40)	7.90 (2.68)	0.09
Hematocrit (%)	25.27 (6.94)	24.61 (7.75)	0.09
Platelets (10^3^/μl)	115.88 (98.84)	116.01 (97.93)	0.00
Creatinine (μmol/l)	106.70 (84.04)	111.06 (75.33)	0.06
Sodium (mmol/L)	129.74 (6.67)	129.55 (6.26)	0.03
K (μmol/l)	4.00 (0.98)	4.01 (0.86)	0.01
AST (units/l)	200.84 (209.80)	162.71 (180.44)	0.20
Lower respiratory infection (yes)	23 (32.9)	21 (30.0)	0.06
CNS infection (yes)	7 (10.0)	6 (8.6)	0.05
Fungemia (yes)	69 (98.6)	68 (97.1)	0.10
LOS (days)	17.29 (11.39)	15.14 (8.57)	0.21

*Note*: Summary statistics are presented as count (percentage) of positive value for categorical variables and mean (standard deviation) for continuous variables. SMD: standardized mean difference, n: group sample size, IVDU: intravenous drug use, CNS: central nervous system, AST: aspartate aminotransferase, TB: tuberculosis, TM: *Talaromyces marneffei*, SBP: systolic blood pressure, K: potassium, LOS: length of stay.

Table 3 shows the results of the Bayesian multivariate logistic regression to estimate the average treatment effects following the matching of the 242 patients in the two treatment groups. The odds ratio for poor outcomes on day 14 for patients treated with amphotericin relative to itraconazole was 1.25 (95% CI: 0.62–2.50). In a sensitivity analysis using the larger sample set (n = 421), the odds of poor outcomes for patients treated with amphotericin relative to itraconazole was 1.69 (95% CI: 0.92–3.13) (Supplementary Table 5). An additional sensitivity analysis using a caliper of 0.2 standard deviations during matching resulted in an OR of 1.04 (95% CI: 0.48–2.22) (Supplementary Table 6).

**Table 3.** Results of the Bayesian multivariate logistic regression of the risk of poor outcomes at day 14 for treatment with amphotericin versus itraconazole on the matched cohort (n = 242).

	Odds ratio	2.5% CI	97.5% CI
Sex (male)	1.33	0.59	2.98
IVDU (yes)	1.94	0.93	4.05
Fever (>38°C)	0.66	0.31	1.37
Pulse (beats/minute)	1.00	0.96	1.03
Respiratory rate (breaths/minute)	1.08	0.98	1.20
Skin lesions (yes)	0.90	0.44	1.86
Absolute lymphocyte count (cells/μl)	1.43	0.86	2.51
Hemoglobin (g/dldl)	0.93	0.77	1.12
Platelets (10^3^/μ)	0.99	0.99	1.00
Creatinine (μmol/l)	1.00	1.00	1.01
AST (units/l)	1.00	1.00	1.01
Lower respiratory infection (yes)	1.17	0.57	2.39
CNS infection (yes)	1.24	0.54	2.88
Fungemia (yes)	1.14	0.45	2.91
Treatment (amphotericin B)	1.25	0.62	2.50

*Note*: AST: aspartate aminotransferase, CNS: central nervous system, IVDU: intravenous drug use.

## Discussion

Our prognostic model identified history of IVDU, absence of fever, higher respiratory rate, higher AST, higher creatinine, higher absolute lymphocyte count and a concurrent pneumonia or CNS infection as independent predictors of poor outcomes. Only IVDU, fever, respiratory rate and lymphocyte count were identified as predictors of poor outcomes in the original analysis of this patient cohort by Le et al., because that study focused on a pre-determined smaller set of covariates.^1^ With the exception of lymphocyte count, the prognostic factors identified in our study have been associated with outcomes in HIV and other HIV-associated mycoses. Dyspnea on admission has been associated with mortality in another talaromycosis cohort in Vietnam^6^ and in an HIV-associated histoplasmosis cohort in French Guiana.^27^ Elevated liver transaminases due to viral hepatitis coinfection were predictive of poor HIV prognosis in a systematic review.^28^ Elevated creatinine is associated with poor clinical outcomes and mortality in HIV disease due to HIV-associated nephropathy.^16^^,^^29^ Fever, interestingly, is a good prognostic factor. This is likely because fever triggers a more comprehensive diagnostic work up including blood culture. Blood culture provides the mainstay of diagnosis of talaromycosis, particularly in patients without skin lesions, enabling treatment which reduces mortality. We found that patients with poor outcomes were less likely to present with skin lesions; however, the odds ratio was not statistically significant in our prognostic model (Table 1). A recent prognostic model for HIV-associated tuberculosis found CD4 count and antiretroviral therapy (ART) status to be important predictors of patient outcomes.^30^ We used absolute lymphocyte count as a surrogate of CD4 count, as CD4 count was not routinely performed in the inpatient setting. The finding that higher absolute lymphocyte count was associated with poor outcomes is unexpected and should be validated. Elevated lymphocyte count is associated with chronic infections, cancers and autoimmune or inflammatory diseases. Such comorbidities are prevalent syndemics of HIV. Further research to understand the dynamics of this relationship and determine whether it is an artifact of the data or a useful biomarker of talaromycosis or its comorbidities may be worthwhile. We did not include ART in our model as only 20% of patients were on ART at presentation during the study period.^1^ We also did not include sepsis in our model, as this comorbidity was not a strong independent predictor of patient outcomes. Their inclusion did not have any appreciable impact on the estimates of patient outcomes; therefore, we excluded these variables to achieve a more parsimonious model.

This study differs substantially from the prior analysis of this cohort. The focus is on developing and validating a prognostic model to accurately predict outcomes when presented with a patient suspected of talaromycosis in a clinical setting.^20^ Our prognostic model exhibited good discrimination for both in-sample (AUROC of 0.85) and out-of-sample (AUROC of 0.91) validation (Fig. 1A, B). There was no decrease in model performance between in-sample and out-of-sample validation, which should allay most concerns about the potential of overfitting of the training data. The higher out-of-sample AUROC may be due to the smaller sample size of the validation set, with the test data somehow more favorable to the model. Overall, the prognostic performance did not exceed that of the APACHE model,^12^ but did exceed that of the HIV-associated tuberculosis model^30^ and should be further externally validated and refined.

This study is novel in the assessment of treatment effect using propensity score matching in order to draw comparisons with the results of the IVAP trial. The causal model used propensity scores to identify subsets of the data which could be paired to emulate randomization. This was done in an effort to exploit the unique opportunity of having results from a randomized control trial to validate the results of observational data using the matching technique. The causal model showed that, compared to itraconazole, induction therapy with amphotericin B had an OR of a poor outcome of 1.25, with a 95% credible interval between 0.62 and 2.50 (Table 3). In the IVAP trial, the primary outcome was 2-week mortality, and the absolute risk difference between amphotericin and itraconazole was 0.9 (95% confidence interval: −3.9 to 5.6).^10^ While the methodologies used differ, the results of our causal model are similar to the results obtained in the IVAP trial. The findings were consistent with the results of a sensitivity analysis in which minimal exclusion criteria were applied to the data, as well as when a caliper was applied to enforce a ceiling on the distance between potential matches. This suggests agreement with the IVAP trial results that there was no substantial difference in patient outcome given induction therapy with itraconazole or amphotericin in the short term (over 2 weeks). We were unable to investigate long-term outcomes in this study due to lack of follow-up in this dataset. But the IVAP trial showed that mortality at 6 months was 50% lower in patients receiving amphotericin B, along with faster fungal clearance from blood and lower risks of relapse, which distinguishes amphotericin B as a superior induction therapy for talaromycosis.^10^ As antifungal therapy for invasive fungal infections requires a prolonged treatment period of up to 12 months, these studies emphasize the importance of conducting longer follow up for accurate assessment of treatment effects for talaromycosis and other HIV-associated invasive mycoses.

Our study has limitations due to the retrospective nature of the data. The propensity score matching procedure for the causal model generally yielded reasonable results, but the distribution of propensity scores after matching were different in the upper tail, with the itraconazole group exhibiting fewer observations with high propensity for assignment to the amphotericin B group (Supplementary Fig. 1). This means difficulty in matching some patients who received amphotericin B with patients who had a similarly high predicted propensity to be prescribed amphotericin based on our model, but instead received itraconazole. Baseline variables such as body mass index (BMI) and additional comorbidities beyond the bloodstream, CNS and respiratory infections which we have analyzed, if they are available, may have improved the propensity score matching procedure. The IVAP study showed that the colony forming units of *T. marneffei* in the blood were a strong quantitative indicator of disease severity and predictor of mortality.^10^ This variable was not available for this study and should be incorporated in future prognostic studies. Finally, the SMD of the background variables after matching did not always fall below the heuristic of 0.1. Using a caliper improved these results for some covariates, though at the expense of sample size (Supplementary Table 4). Although this is only a heuristic, the findings limit the strength of the conclusions we may draw.

Notwithstanding the aforementioned limitations, these results meet our goal to develop a clinical decision support tool to aid physicians in understanding the interplay between common clinical findings and disease outcomes in a real-life cohort of talaromycosis patients. While the prognostic model should be improved upon, the results represent an important step in our effort to quantitatively assess disease severity as patients present to the hospital. Our next step is to externally validate and further refine the prognostic model using prospectively collected data, including additional covariates from multiple clinical sites in Southeast Asia. Our goal is to develop a scoring application, which can be accessed via a computer or a smartphone, to aid clinicians in the assessment of disease severity and in the triage and management of this life-threatening but neglected tropical mycosis. We also demonstrate concurrence in treatment effects at 2 weeks between a real-world patient cohort and the previously published IVAP trial cohort using a causal model, providing a useful analytical frame work for other investigators to evaluate treatment effects in future observational studies.

## Supplementary Material

myab005_Supplementary_File
