# Supplementary material for: Prognosis and treatment effects of HIV-associated talaromycosis in a real-world patient cohort
Source: Med Mycol. 2021 Feb 27;59(4):392–9. doi: 10.1093/mmy/myab005 (PMC8023982; doi:10.1093/mmy/myab005)
Supplement: myab005_Supplementary_File [file myab005_supplementary_file.docx]

# Supporting information

**Table S1. Pre-matching assessment of baseline variables for amphotericin B and itraconazole treatment groups with main exclusion criteria applied to the data (n=421)**

|  | Amphotericin B | Itraconazole | SMD |
| --- | --- | --- | --- |
| n | 109 | 312 | - |
| Age (years) | 28.48 (4.99) | 28.85 (6.11) | 0.07 |
| Sex (male) | 85 (78.0) | 254 (81.4) | 0.09 |
| IVDU (yes) | 66 (60.6) | 214 (68.6) | 0.17 |
| History of TB (yes) | 31 (28.4) | 77 (24.7) | 0.09 |
| History of TM (yes) | 6 (5.5) | 16 (5.1) | 0.02 |
| Fever (> 38 C) | 42 (38.5) | 99 (31.7) | 0.14 |
| Pulse (beats/min) | 101.04 (17.54) | 99.62 (17.06) | 0.08 |
| SBP (mm Hg) | 99.63 (12.22) | 97.75 (13.59) | 0.15 |
| Respiratory rate (breaths/minute) | 27.26 (6.37) | 26.04 (6.23) | 0.19 |
| Weight (kg) | 44.20 (7.16) | 43.21 (6.87) | 0.14 |
| Skin lesions (yes) | 52 (47.7) | 264 (84.6) | 0.85 |
| Absolute lymphocyte count (cells/uL) | 0.61 (0.86) | 0.59 (0.75) | 0.02 |
| Hemoglobin (g/dL) | 8.37 (2.32) | 7.52 (2.62) | 0.34 |
| Hematocrit (%) | 26.06 (6.74) | 23.50 (7.48) | 0.36 |
| Platelets (10^3^/uL) | 104.36 (101.32) | 118.90 (107.93) | 0.14 |
| Creatinine (umol/L) | 104.64 (70.53) | 96.54 (54.32) | 0.13 |
| Sodium (mmol/L) | 129.75 (6.19) | 130.61 (6.31) | 0.14 |
| K (umol/L) | 4.01 (0.91) | 3.74 (0.79) | 0.31 |
| AST (units/L) | 214.54 (197.49) | 134.44 (145.19) | 0.46 |
| Lower respiratory infection (yes) | 36 (33.0) | 96 (30.8) | 0.05 |
| CNS infection (yes) | 11 (10.1) | 10 (3.2) | 0.28 |
| Fungemia (yes) | 108 (99.1) | 288 (92.3) | 0.34 |
| LOS (days) | 13.56 (11.15) | 13.38 (10.66) | 0.02 |

*Note*: Summary statistics presented above are count (percentage) of positive value for categorical variables and mean (standard deviation) for continuous variables. SMD: standardized mean difference, n: group sample size, IVDU: intravenous drug use, CNS: central nervous system, AST: aspartate aminotransferase, TB: tuberculosis, TM: *Talaromyces marneffei*, SBP: systolic blood pressure, K: potassium, LOS: length of stay.

**Table S2.** **Pre-matching assessment of baseline variables for amphotericin B and itraconazole treatment groups with additional IVAP exclusion criteria applied to the data (n=242)**

|  | Amphotericin B | Itraconazole | SMD |
| --- | --- | --- | --- |
| n | 70 | 172 | - |
| Age (years) | 28.79 (4.71) | 28.42 (5.71) | 0.07 |
| Sex (male) | 54 (77.1) | 141 (82.0) | 0.12 |
| IVDU (yes) | 40 (57.1) | 125 (72.7) | 0.33 |
| History of TB (yes) | 23 (32.9) | 40 (23.3) | 0.22 |
| History of TM (yes) | 5 (7.1) | 7 (4.1) | 0.13 |
| Fever (> 38 C) | 26 (37.1) | 56 (32.6) | 0.10 |
| Pulse (beats/min) | 97.63 (16.51) | 99.03 (15.48) | 0.09 |
| SBP (mm Hg) | 100.14 (11.73) | 97.30 (12.60) | 0.23 |
| Respiratory rate (breaths/minute) | 25.74 (5.10) | 25.55 (5.78) | 0.04 |
| Weight (kg) | 44.61 (7.64) | 43.28 (6.75) | 0.18 |
| Skin lesions (yes) | 31 (44.3) | 151 (87.8) | 1.03 |
| Absolute lymphocyte count (cells/uL) | 0.59 (0.93) | 0.51 (0.61) | 0.10 |
| Hemoglobin (g/dL) | 8.14 (2.40) | 7.57 (2.53) | 0.23 |
| Hematocrit (%) | 25.27 (6.94) | 23.74 (7.23) | 0.22 |
| Platelets (10^3^/uL) | 115.88 (98.84) | 118.46 (99.08) | 0.03 |
| Creatinine (umol/L) | 106.70 (84.04) | 97.98 (57.78) | 0.12 |
| Sodium (mmol/L) | 129.74 (6.67) | 130.37 (6.12) | 0.10 |
| K (umol/L) | 4.00 (0.98) | 3.71 (0.77) | 0.34 |
| AST (units/L) | 200.84 (209.80) | 124.81 (139.11) | 0.43 |
| Lower respiratory infection (yes) | 23 (32.9) | 45 (26.2) | 0.15 |
| CNS infection (yes) | 7 (10.0) | 8 (4.7) | 0.21 |
| Fungemia (yes) | 69 (98.6) | 159 (92.4) | 0.30 |
| LOS (days) | 17.29 (11.39) | 15.55 (9.81) | 0.16 |

*Note*: Summary statistics presented above are count (percentage) of positive value for categorical variables and mean (standard deviation) for continuous variables. SMD: standardized mean difference, n: group sample size, IVDU: intravenous drug use, CNS: central nervous system, AST: aspartate aminotransferase, TB: tuberculosis, TM: *Talaromyces marneffei*, SBP: systolic blood pressure, K: potassium, LOS: length of stay.

**Table S3.** **Post-matching assessment of baseline variables for amphotericin B and itraconazole treatment groups with main exclusion criteria applied to the data (n=421)**

|  | Amphotericin B | Itraconazole | SMD |
| --- | --- | --- | --- |
| n | 109 | 109 | - |
| Age (years) | 28.48 (4.99) | 28.89 (5.82) | 0.08 |
| Sex (male) | 85 (78.0) | 87 (79.8) | 0.05 |
| IVDU (yes) | 66 (60.6) | 68 (62.4) | 0.04 |
| History of TB (yes) | 31 (28.4) | 28 (25.7) | 0.06 |
| History of TM (yes) | 6 (5.5) | 5 (4.6) | 0.04 |
| Fever (> 38 C) | 42 (38.5) | 38 (34.9) | 0.08 |
| Pulse (beats/min) | 101.04 (17.54) | 101.67 (16.87) | 0.04 |
| SBP (mm Hg) | 99.63 (12.22) | 97.50 (13.78) | 0.16 |
| Respiratory rate (breaths/minute) | 27.26 (6.37) | 26.69 (7.07) | 0.08 |
| Weight (kg) | 44.20 (7.16) | 43.24 (6.26) | 0.14 |
| Skin lesions (yes) | 52 (47.7) | 62 (56.9) | 0.18 |
| Absolute lymphocyte count (cells/uL) | 0.61 (0.86) | 0.69 (0.80) | 0.10 |
| Hemoglobin (g/dL) | 8.37 (2.32) | 8.37 (2.86) | 0.00 |
| Hematocrit (%) | 26.06 (6.74) | 25.93 (8.13) | 0.02 |
| Platelets (10^3^/uL) | 104.36 (101.32) | 105.27 (88.86) | 0.01 |
| Creatinine (umol/L) | 104.64 (70.53) | 107.19 (68.55) | 0.04 |
| Sodium (mmol/L) | 129.75 (6.19) | 130.01 (6.46) | 0.04 |
| K (umol/L) | 4.01 (0.91) | 3.93 (0.87) | 0.09 |
| AST (units/L) | 214.54 (197.49) | 177.51 (203.37) | 0.19 |
| Lower respiratory infection (yes) | 36 (33.0) | 36 (33.0) | <0.001 |
| CNS infection (yes) | 11 (10.1) | 7 (6.4) | 0.13 |
| Fungemia (yes) | 108 (99.1) | 108 (99.1) | <0.001 |
| LOS (days) | 13.56 (11.15) | 14.82 (13.10) | 0.10 |

*Note*: Summary statistics presented above are count (percentage) of positive value for categorical variables and mean (standard deviation) for continuous variables. SMD: standardized mean difference, n: group sample size, IVDU: intravenous drug use, CNS: central nervous system, AST: aspartate aminotransferase, TB: tuberculosis, TM: *Talaromyces marneffei*, SBP: systolic blood pressure, K: potassium, LOS: length of stay.

**Table S4.** **Post-matching assessment of baseline variables for amphotericin B and itraconazole treatment groups with additional IVAP exclusion criteria and a caliper of 0.2 standard deviations applied to the data (n=242)**

|  | Amphotericin B | Itraconazole | SMD |
| --- | --- | --- | --- |
| n | 49 | 49 | 0.00 |
| Age (years) | 28.88 (4.75) | 28.43 (5.75) | 0.09 |
| Sex (male) | 40 (81.6) | 38 ( 77.6) | 0.10 |
| IVDU (yes) | 29 (59.2) | 30 ( 61.2) | 0.04 |
| History of TB (yes) | 16 (32.7) | 13 ( 26.5) | 0.13 |
| History of TM (yes) | 5 (10.2) | 3 ( 6.1) | 0.15 |
| Fever (> 38 C) | 19 (38.8) | 17 ( 34.7) | 0.09 |
| Pulse (beats/min) | 99.43 (16.27) | 96.49 (16.94) | 0.18 |
| SBP (mm Hg) | 102.04 (10.80) | 97.86 (10.21) | 0.40 |
| Respiratory rate (breaths/minute) | 24.82 (4.48) | 25.10 (4.78) | 0.06 |
| Weight (kg) | 45.61 (7.58) | 42.73 (7.96) | 0.37 |
| Skin lesions (yes) | 31 (63.3) | 31 ( 63.3) | <0.001 |
| Absolute lymphocyte count (cells/uL) | 0.68 (1.08) | 0.67 (0.95) | 0.02 |
| Hemoglobin (g/dL) | 7.95 (2.34) | 7.83 (2.48) | 0.05 |
| Hematocrit (%) | 24.84 (6.79) | 24.36 (7.30) | 0.07 |
| Platelets (10^3^/uL) | 118.88 (100.20) | 120.34 (104.51) | 0.01 |
| Creatinine (umol/L) | 110.47 (92.63) | 108.18 (63.34) | 0.03 |
| Sodium (mmol/L) | 130.01 (6.76) | 129.68 (6.14) | 0.05 |
| K (umol/L) | 3.88 (1.04) | 3.90 (0.79) | 0.02 |
| AST (units/L) | 177.45 (188.71) | 149.94 (198.14) | 0.14 |
| Lower respiratory infection (yes) | 16 (32.7) | 14 (28.6) | 0.09 |
| CNS infection (yes) | 5 (10.2) | 5 (10.2) | <0.001 |
| Fungemia (yes) | 48 (98.0) | 47 (95.9) | 0.12 |
| LOS (days) | 18.67 (12.58) | 15.65 (8.96) | 0.28 |

*Note*: Summary statistics presented above are count (percentage) of positive value for categorical variables and mean (standard deviation) for continuous variables. SMD: standardized mean difference, n: group sample size, IVDU: intravenous drug use, CNS: central nervous system, AST: aspartate aminotransferase, TB: tuberculosis, TM: *Talaromyces marneffei*, SBP: systolic blood pressure, K: potassium, LOS: length of stay.

**Table S5.** **Results of causal model when only the main exclusion criteria were applied to the data (n=421)**

|  | | | |
| --- | --- | --- | --- |
|  | Odds Ratio | 2.5% CI | 97.5% CI |
| Sex (male) | 1.50 | 0.72 | 3.17 |
| IVDU (yes) | 2.07 | 1.07 | 4.07 |
| Fever (> 38 C) | 0.47 | 0.24 | 0.91 |
| Pulse (beats/minute) | 1.01 | 0.98 | 1.03 |
| Respiratory rate (breaths/minute) | 1.12 | 1.06 | 1.19 |
| Skin lesions (yes) | 1.08 | 0.59 | 1.98 |
| Absolute lymphocyte count (cells/uL) | 1.85 | 1.12 | 3.19 |
| Hemoglobin (g/dL) | 0.90 | 0.77 | 1.04 |
| Platelets (10^3^/uL) | 0.99 | 0.99 | 1.00 |
| Creatinine (umol/L) | 1.00 | 1.00 | 1.01 |
| AST (units/L) | 1.00 | 1.00 | 1.01 |
| Lower respiratory infection (yes) | 0.88 | 0.47 | 1.65 |
| CNS infection (yes) | 1.68 | 0.77 | 3.65 |
| Fungemia (yes) | 1.09 | 0.41 | 2.81 |
| Treatment (amphotericin B) | 1.69 | 0.92 | 3.13 |

*Note*: Coefficients and credible intervals reflect the odds of poor outcomes at 14 days. CI: credible interval, IVDU: intravenous drug use, CNS: central nervous system, AST: aspartate aminotransferase.

**Table S6.** **Results of causal model when additional IVAP exclusion criteria and a caliper of 0.2 standard deviations were applied to the data (n=242)**

|  | | | |
| --- | --- | --- | --- |
|  | Odds Ratio | 2.5% CI | 97.5% CI |
| Sex (male) | 1.15 | 0.50 | 2.73 |
| IVDU (yes) | 1.89 | 0.85 | 4.28 |
| Fever (> 38 C) | 0.89 | 0.39 | 2.03 |
| Pulse (beats/minute) | 1.00 | 0.96 | 1.05 |
| Respiratory rate (breaths/minute) | 1.07 | 0.93 | 1.24 |
| Skin lesions (yes) | 1.31 | 0.59 | 2.88 |
| Absolute lymphocyte count (cells/uL) | 1.67 | 0.95 | 3.19 |
| Hemoglobin (g/dL) | 0.94 | 0.73 | 1.22 |
| Platelets (10^3^/uL) | 1.00 | 0.99 | 1.00 |
| Creatinine (umol/L) | 1.00 | 0.99 | 1.01 |
| AST (units/L) | 1.00 | 1.00 | 1.00 |
| Lower respiratory infection (yes) | 1.05 | 0.47 | 2.31 |
| CNS infection (yes) | 1.35 | 0.56 | 3.23 |
| Fungemia (yes) | 0.93 | 0.36 | 2.41 |
| Treatment (amphotericin B) | 1.04 | 0.48 | 2.22 |

*Note*: IVDU: intravenous drug use, CNS: central nervous system,

AST: aspartate aminotransferase.

**Figure S1. Distribution of matched and unmatched propensity scores following application of the matching procedure**. All patients from the treatment group (amphotericin) were matched with exactly one patient from the control group (itraconazole). **Fig.** **S1 (a)** Post-matching distribution of propensity scores when additional IVAP exclusion criteria were applied to the data. **Fig.** **S1 (b)** Post-matching distribution of propensity scores when only main exclusion criteria were applied to the data.


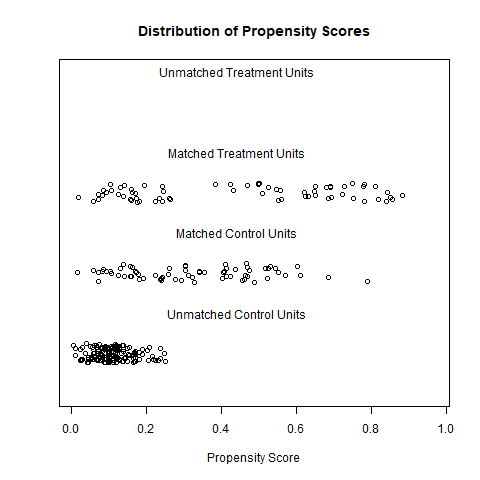

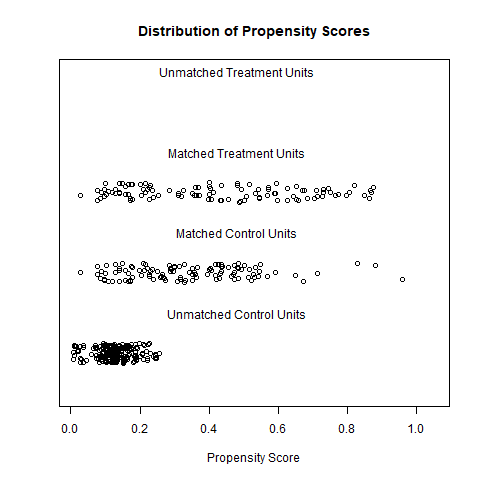


**(a)**

**(b)**
